# Northeastern Chinese Sauerkraut Juice as a Water Replacer in Pork Fillings: Flavor Profiles and Sensory Attributes

**DOI:** 10.3390/foods15193443

**Published:** 2026-09-26

**Authors:** Dezhuang Gong, Mengzhou Wang, Chunling Li, Zhitang Liang, Danshi Zhu

**Affiliations:** 1 National & Local Joint Engineering Research Center of Storage, College of Food Science and Technology, Bohai University, Jinzhou, 121013, China; 2 Foshan Shunde Junling Kitchenware Co., Ltd., Foshan 528000, China

**Keywords:** volatile flavor, Sauerkraut juice, pork baozi fillings, sensory evaluation

## Abstract

In this study, the volatile organic compound (VOC) profiles and sensory quality of pork baozi fillings prepared with different substitution levels of Northeastern Chinese sauerkraut juice were characterized using an electronic nose, electronic tongue, gas chromatography–mass spectrometry (GC–MS), and gas chromatography–ion mobility spectrometry (GC–IMS). The results showed that both the VOC profiles and sensory attributes of pork baozi fillings varied significantly across sauerkraut juice substitution levels. A total of 80 and 66 VOCs were identified by GC–MS and GC–IMS, respectively. Notably, pork baozi fillings with the 75% sauerkraut juice substitution level exhibited the highest VOC diversity, the highest total VOC content, and the highest overall sensory score. Compared with the control group (12,348.57 μg/kg), the total VOC content increased significantly by 155% to 31,495.61 μg/kg at 75% substitution. Orthogonal partial least squares discriminant analysis (OPLS–DA) screened out 19 key differential VOCs, including (E,E)-2,4-decadienal, (E)-2-dodecenal, (E)-9-octadecenoic acid ethyl ester, oleic acid and 2,4-hexadienoic acid, ethyl ester. Seventeen fatty acids were quantified, among which (E)-9-octadecenoic acid (C18:1n9t) and palmitic acid (C16:0) accounted for 55.90% and 21.78% of the total fatty acid content, respectively. Partial replacement of water with sauerkraut juice effectively reduced off-flavors in pork baozi fillings and increased the proportion of unsaturated fatty acids, thus improving overall palatability and nutritional quality. This study provides preliminary insights into the flavor modulation mechanism of sauerkraut juice in pork baozi fillings and offers a theoretical basis for the development of high-quality sauerkraut pork products.

## 1. Introduction

Northeastern Chinese sauerkraut is a traditional low-salt fermented vegetable valued for its unique flavor profile and favourable storability. During fermentation, autochthonous lactic acid bacteria convert cabbage carbohydrates into organic acids, predominantly lactic acid, together with minor amounts of citric, acetic, malic and succinic acids, as well as alcohols, aldehydes and ketones that collectively endow the product with a mild, pleasant sour taste. Meanwhile, most dietary fiber and mineral components are well retained throughout fermentation [1,2]. As a major by-product, sauerkraut juice is rich in bioactive compounds including organic acids, phenolics, glucosinolates, minerals, and vitamin C, with well-documented antioxidant and flavor-enhancing properties. Organic acids are known to preserve the nutritional and sensory quality of food matrices and are widely applied as natural preservatives [3]. When applied to fresh meat, they help maintain physicochemical stability, antioxidant capacity, and microbial safety [4,5]. Unlike single purified organic acids (e.g., lactic acid, acetic acid) commonly used for pH adjustment and microbial inhibition in meat products, sauerkraut juice is a natural complex matrix containing mixed organic acids, phenolics, glucosinolate degradation products and amino acids, which simultaneously modify the volatile and taste profiles of pork baozi fillings [6]. Accordingly, sauerkraut juice shows promising potential as a natural additive to improve both the quality and flavor of meat products.

Meat products provide all essential amino acids in an optimal ratio, as well as long-chain fatty acids, conjugated linoleic acids, nucleotides, and bioactive hydrolysates [7]. Comminuted meat products, formulated through chopping and emulsification of raw meat followed by thermal processing, together with ingredients such as added proteins, fat, water and salt, are widely consumed for their tender texture and high nutritional value [8]. The typical flavor profile of meat is formed through a cascade of complex chemical reactions involving endogenous nutritional components during thermal processing, among which the Maillard reaction, lipid oxidation and carbohydrate degradation serve as the three primary pathways [9]. However, undesirable reactions such as excessive lipid oxidation and protein degradation often occur, generating off-flavor compounds including aldehydes, sulfur-containing volatiles, and amines. These deteriorative reactions mask the inherent meaty aroma and produce rancid, fishy, and other unpleasant notes, markedly reducing sensory quality. The high organic acid content in sauerkraut juice can mitigate such off-flavors. Its deodorizing effect arises from the neutralization of acidic constituents with alkaline nitrogenous compounds in meat, while flavor improvement is attributed to esterification reactions between acids and endogenous alcohols [10]. Furthermore, sauerkraut juice may enrich the flavor of pork baozi fillings not only by enhancing the intensity of existing volatiles but also by promoting the formation of novel flavor compounds through synergistic interactions.

Baozi, a traditional yeast-fermented filled steamed bun, is one of the most widely consumed staple foods in China. During steaming, heat and mass transfer drive flavor migration within the filling matrix, imparting desirable tenderness, juiciness, and aromatic attributes while balancing nutrition and taste. The classic Northeastern Chinese sauerkraut-pork filling, renowned for its fresh, savory, and distinct regional flavor profile, is highly popular among consumers. Prior research on Chinese steamed stuffed bun products has mainly focused on dough fermentation characteristics and textural properties of fillings. For flavor-related studies, existing GC-MS analyses of steamed cereal products have largely centered on the fermented dough matrix, while volatile profiling of pork fillings has mostly been conducted under dry-heat cooking conditions [11]. Specifically, regarding sauerkraut application in meat products, current reports are restricted to sausage, surimi and raw meat preservation systems, and the dose-dependent flavor effects of sauerkraut juice as a water replacer in steamed pork baozi fillings remain unreported. However, both sauerkraut dosage and pretreatment protocols exert a substantial impact on the overall quality of pork baozi fillings. In contrast, sauerkraut juice offers a simpler alternative that can directly replace water during pork baozi fillings preparation. While the organic acids in sauerkraut juice effectively mask undesirable meat-derived off-odors, the specific effects of different replacement ratios on the flavor profile of pork baozi fillings remain unclear. Therefore, the objective of this study was to systematically evaluate the impacts of varying Northeastern Chinese sauerkraut juice replacement ratios on the volatile organic compound (VOC) profiles, key flavor-active compounds, and sensory attributes of pork baozi fillings, so as to clarify the flavor modulation mechanism of sauerkraut juice in steamed meat systems and provide a theoretical basis for the industrial production of high-quality sauerkraut-pork baozi.

## 2. Materials and Methods

### 2.1. Materials

All chemicals and reagents used in this study were of analytical grade unless otherwise specified. Northeastern Chinese sauerkraut was purchased from Yunshanban Flagship Store. Fresh pork and edible condiments were obtained from a local market in Jinzhou, China. The 2-methyl-3-heptanone standard and 14% boron trifluoride-methanol solution were obtained from Macklin Biochemical Technology Co., Ltd. (Shanghai, China). The C4–C9 n-alkyl ketone mixture (purity > 97.0%) was acquired from Sinopharm Group Chemical Reagent Co., Ltd. (Shanghai, China). Sodium chloride (NaCl) and n-hexane were supplied by Damao Chemical Reagent Factory (Tianjin, China). Trichloroacetic acid (TCA), benzene, and petroleum ether were provided by Tianli Chemical Reagent Co., Ltd. (Tianjin, China).

### 2.2. Preparation of Minced Meat and Sauerkraut Juice

Pork was ground through an 8 mm aperture plate using a meat grinder (TJ12H, Guangzhou Henglian Food Machinery Co., Ltd., Guangzhou, China) and stored at −18 °C. Sauerkraut juice was extracted by direct pressing, filtered through a 300-mesh filter cloth, and stored at 4 °C for a maximum of 48 h before use. The raw sauerkraut juice had a pH of 3.44 ± 0.02, a titratable acidity of 11.9 ± 0.3 g/kg, a total phenolic content of 0.111 ± 0.002 mg/mL, and a sodium content of 781 ± 21 mg/100 g.

### 2.3. Processing of Baozi

Five groups of baozi were prepared with different sauerkraut juice substitution levels for water. The control group (0% substitution) used pure deionized water as the liquid component, while four experimental groups were set with substitution levels of 25%, 50%, 75%, and 100%, respectively.

Fatty meat and lean meat were mixed at a ratio of 3:7. The mixture was homogenized using a food processor (Hauswirt M5, Hanshang Electrical Appliance Co., Ltd., Qingdao, China) at 90 rpm for 1 min. Seasonings including 2% oyster sauce, 0.6% salt, 3% light soy sauce, and 3% dark soy sauce (all percentages based on total meat weight) were added, followed by blending for another 1 min. A total of 32% liquid (deionized water and/or sauerkraut juice) was incorporated gradually in four equal aliquots, with vigorous stirring for 30 s after each addition. Subsequently, 0.6% monosodium glutamate and 0.2% five-spice powder were added and mixed at 90 rpm for 30 s. Finally, 6% soybean oil was added and blended at the same speed for an additional 30 s to obtain raw pork fillings.

Each baozi was made by enclosing 20 g of raw filling in 30 g of dough, which was then kneaded thoroughly and proofed at 37 °C and 75% relative humidity for 40 min. After proofing, samples were stored at −80 °C. Prior to analysis, frozen baozi were steamed in boiling water for 20 min. Steamed pork fillings separated from baozi (defined as pork baozi fillings) were used for all experiments. A detailed flowchart of the manufacturing process is shown in Figure 1. The pH values of the final steamed pork baozi fillings were first determined to characterize the acidic gradient induced by sauerkraut juice substitution. The pH values were 6.80 ± 0.02, 6.42 ± 0.03, 5.97 ± 0.04, 5.63 ± 0.03, and 5.42 ± 0.04 for the 0%, 25%, 50%, 75%, and 100% substitution groups, respectively.

### 2.4. GC–MS Analysis

The SPME-GC-MS method was adapted from [12] with minor modifications. Briefly, 1 g of finely ground pork baozi filling sample was transferred into a 20 mL headspace vial, supplemented with 2 mL saturated NaCl solution and 20 μL 2-methyl-3-heptanone solution (81.6 mg/L, used as internal standard), and the vial was immediately hermetically sealed. A 75 μm CAR/PDMS fiber (Supelco, Bellefonte, PA, USA) was applied for Solid-phase microextraction (SPME) extraction: the sample vial was first equilibrated at 45 °C for 10 min, and then the fiber was exposed to the headspace for 30 min to adsorb VOCs. Subsequently, the fiber was withdrawn and inserted into the GC injection port for 5 min of thermal desorption at 250 °C, and the analytes were separated and detected using a TQ-8040 NX GC-MS system (Shimadzu Co., Ltd., Kyoto, Japan).

The oven temperature program was set as follows: initial temperature 40 °C (held for 3 min), increased to 100 °C at 3 °C/min, then raised to 230 °C and held for 5 min. Helium was used as the carrier gas at a constant flow rate of 1.0 mL/min. The mass spectrometric conditions were: ion source temperature 230 °C, solvent delay 2.5 min, and scan range 35–500 *m*/*z*. Semi-quantitative analysis was carried out using 2-methyl-3-heptanone as the internal standard. The detailed calculation formula is presented below.

$$C_{i}=\frac{S_{i}\times M_{0}}{S_{0}\times M_{i}}\times 1000$$

where *C_i_* denotes the content of the identified compound (μg/kg); *S*_0_ and *S_i_* denote the peak areas of the internal standard and the identified compound, respectively; *M*_0_ represents the mass of the added 20 μL internal standard solution (μg); *M_i_* is the mass of the pork baozi filling sample (g).

### 2.5. GC-IMS Analysis

Volatile organic compounds (VOCs) in all samples were analyzed using a FlavorSpec^®^ GC-IMS instrument (Dortmund, Germany). The method was adapted from [13] with minor modifications. Briefly, 1 g of finely ground pork baozi filling sample was weighed into a 20 mL headspace vial and incubated at 60 °C for 15 min with 500 rpm agitation. Afterwards, 500 μL of the headspace gas was withdrawn and injected into the GC inlet. Separation was performed on an FS-SE-54-CB-1 capillary column (15 m × 0.53 mm × 1.0 μm film thickness). Nitrogen was used as both the carrier gas and drift gas. The carrier gas flow program was as follows: 2 mL/min for 2 min, linearly increased to 10 mL/min over 8 min, then to 100 mL/min over the next 10 min, and held at 100 mL/min for a further 10 min (total run time: 30 min). The drift gas flow rate was maintained at a constant 150 mL/min throughout the analysis. Retention indices were determined using a C4–C9 n-alkyl ketone mixture as external calibration references, and compound identification was performed by matching their retention indices and migration times against the built-in VOICE database (G.A.S., Dortmund, Germany). The built-in Reporter plugin was used to generate 2D and 3D topographic plots, while the Gallery Plot plugin was applied to construct VOC fingerprint profiles.

### 2.6. Relative Fatty Acid Content Analysis

Fat extraction and methylation procedures were modified based on the method reported by [14]. Briefly, 50 g of finely ground pork baozi filling sample was blended with 100 mL chloroform-methanol solution (2:1, *v*/*v*, containing 0.01% butylated hydroxytoluene), extracted by soaking, and filtered through qualitative filter paper. Subsequently, saturated sodium chloride solution (20 mL) was added to the obtained filtrate. The mixture was vortexed thoroughly and allowed to stand until complete separation of the aqueous and organic phases was observed. The organic phase located at the bottom was collected, and the lipid fraction therein was concentrated via reduced-pressure rotary evaporation with a water bath set at 45 °C.

Preparation of fatty acid methyl esters (FAMEs): Accurately weighed 50 mg of the extracted lipid was transferred into a clean and dry glass test tube, followed by addition of 2 mL benzene-petroleum ether mixture (1:1, *v*/*v*). The solution was gently swirled to fully disperse the lipid. Then, 2 mL of 14% boron trifluoride-methanol solution was added, and the mixture was fully vortexed. To ensure thorough derivatization, the tube was incubated in a 45 °C water bath for 30 min with intermittent shaking. After incubation, FAMEs were extracted by adding 1 mL n-hexane and an appropriate amount of saturated sodium chloride solution. The mixture was vortexed and allowed to stand until the upper organic phase clarified. The supernatant was filtered through a 0.22 μm organic membrane filter before GC-MS analysis.

GC-MS conditions: The inlet temperature was set at 250 °C. Helium was used as the carrier gas at a constant flow rate of 1.0 mL/min. A 1 μL aliquot was injected in split mode with a split ratio of 20:1. The column oven was initially held at 140 °C for 2 min, heated to 200 °C at a rate of 6 °C/min and maintained for 2 min, then raised to 230 °C at 2 °C/min for another 2-min isothermal hold, and finally ramped up to 250 °C at 4 °C/min with a 2-min hold. For mass spectrometric detection, the interface temperature was set to 250 °C, the electron ionization (EI) source temperature was 200 °C, the solvent delay was 3 min, and the mass scan range was set to 25–500 *m*/*z*. Fatty acids were identified by comparing their mass spectra with the NIST 2020 mass spectral database.

### 2.7. E-Nose Analysis

The aroma profiles of pork baozi filling samples were analyzed using a cNose electronic nose (Baosheng Technology Co., Ltd., Shanghai, China) equipped with a 28-sensor array, according to the method described in reference [13]. Briefly, 10.0 g of finely ground sample was placed in a headspace vial, sealed, and equilibrated in a water bath at 40 °C for 10 min before analysis. The headspace temperature was kept at 40 ± 1 °C throughout detection. The instrumental parameters were set as follows: carrier gas flow rate at 1 L/min, detection time at 60 s, and cleaning time at 120 s. The maximum response value of each sensor was recorded for subsequent multivariate statistical analysis.

### 2.8. E-Tongue Analysis

Taste profiles of pork baozi filling samples were analyzed using an SA402B electronic tongue (Insent Inc., Atsugi-shi, Japan). Briefly, 10.0 g of finely ground sample was diluted with 200 mL deionized water and stirred continuously for 10 h. The mixture was filtered sequentially through 0.45 μm and 0.22 μm membrane filters to obtain a clear supernatant. 35 mL of the clarified filtrate was transferred into a dedicated electronic tongue sample cup and loaded onto the automatic sampler in a predetermined order. The standard measurement cycle proceeded as follows: the sensor array was first cleaned in the proprietary cleaning solution for 90 s, then rinsed in the first reference solution for 120 s, followed by an additional 120 s rinse in the second reference solution. After baseline equilibration for 30 s, the sensors were immersed in the sample for a 30 s measurement cycle, and finally rinsed sequentially in both reference solutions for 3 s each after each measurement. Each sample was measured four consecutive times. The first replicate was discarded to eliminate carryover effects, and the average value of the remaining three replicates was used for subsequent taste component analysis.

### 2.9. Sensory Evaluation

Sensory evaluation of pork baozi fillings was conducted by 30 trained sensory panelists (15 males and 15 females, aged 20–25 years) from the College of Food Science, following a previously described sensory scoring method for meat products [15]. All evaluations were performed in a standardized sensory evaluation laboratory. Samples were coded with random three-digit numbers and presented in a randomized order. Five attributes (appearance, aroma, texture, mouthfeel, and sourness) were assessed using a 20-point scoring scale divided into four score ranges: 16–20, 11–15, 6–10, and 1–5 points. Between sample evaluations, panelists thoroughly rinsed their mouths with purified water and rested for 2 min to avoid sensory fatigue. All evaluations were conducted independently in individual booths with no communication between assessors. The average score of all panelists was calculated as the final sensory result.

High scores (16–20 points) were assigned to samples that met the following criteria: in terms of appearance, the filling maintained structural integrity with no obvious depressions or breakage, and the surface was uniform and lustrous with no noticeable color variation; regarding aroma, the filling exhibited a rich, balanced sour and savory aroma characteristic of pork baozi fillings, with no detectable off-odors; for texture, the filling was densely aggregated without disintegration, and the cross-section was homogeneous and free of large voids or pores; with respect to mouthfeel, the filling was tender and smooth, with high juiciness, moderate chewiness, and a lingering savory aftertaste; and in terms of sourness, the acidity was mild and well-integrated with other flavors, without excessive sharpness or bitterness.

### 2.10. Statistical Analysis

All experiments were performed using three independent biological replicates, corresponding to three separate batches of pork baozi fillings prepared on different days; each biological replicate was measured in analytical triplicate. The results are expressed as mean values ± standard deviation (SD). Significant differences across sample groups were identified via one-way analysis of variance (ANOVA) coupled with Duncan’s multiple range test, and statistical significance was defined at *p* < 0.05. Orthogonal partial least squares discriminant analysis (OPLS-DA) and variable importance in projection (VIP) analyses were performed using SIMCA 14.1 software (Umetrics, Umeå, Sweden), and the resulting plots were visualized therein. Bar charts and heatmaps were generated using Origin 2025 Pro (OriginLab Corporation, Northampton, MA, USA) and the Metware Cloud platform (https://cloud.metware.cn/, accessed on 20 September 2026).

## 3. Results and Discussion

### 3.1. Volatile Flavor Compounds

The VOCs in pork baozi fillings with different sauerkraut juice substitution levels were analyzed by GC–MS. The results showed that sauerkraut juice substitution level significantly affected both the types and concentrations of volatile flavor compounds in the fillings. A total of 80 volatile compounds were identified across all five groups, including 11 esters, 36 aldehydes, 9 ketones, 10 organic acids, and 14 other volatile substances. To visualize the differential distribution and variation patterns of volatile components in pork baozi fillings across different substitution gradients, cluster heatmap analysis was adopted. The number of detected flavor compounds in the 0%, 25%, 50%, 75%, and 100% substitution groups was 46, 54, 64, 66, and 65, respectively, indicating that sauerkraut juice exerts a significant effect on shaping the overall flavor profile of pork baozi fillings. The VOC composition and corresponding cluster heatmap are shown in Figure 2A–C. Notably, both the number and total concentration of VOCs in the fillings peaked at the 75% substitution level. Compared with the control group (12,348.57 μg/kg), the total VOC content increased significantly by 155% to 31,495.61 μg/kg at 75% substitution. During filling preparation, marination with salt and soy sauce enhanced VOC diversity. This was because salt promotes lipid oxidation and exerts a salting-out effect on flavor compounds [16]. Furthermore, sauerkraut juice is inherently rich in VOCs; Wang et al. (2023) identified 148 volatile substances in sauerkraut, including aldehydes, alcohols, esters, and organic acids [17]. However, when the substitution level increased further to 100%, both the number and total content of VOCs decreased to 65 and 22,248.33 μg/kg, respectively. The observed decline may arise because the high dosage of sauerkraut juice introduces substantial phenolic compounds and vitamin C. Such constituents promote secondary reactions among newly formed volatiles, including condensation and polymerization. These reactions consume volatile compounds and reduce the final detectable total VOC content.

Ester compounds are mainly formed through esterification reactions between organic acids and alcohols, which are primarily derived from lipid oxidation and hydrolysis. These compounds generally have relatively high olfactory thresholds and are present at low concentrations in meat, contributing minimally to overall meat flavor [18]. Consistently, the total ester content in the control group pork baozi filling was relatively low at 2663.30 μg/kg. This aligns with the findings of Duan et al. (2023), who detected only five ester compounds in fresh pork using GC-IMS [19]. However, after sauerkraut juice addition, the abundant organic acids in sauerkraut juice underwent esterification with endogenous alcohols in minced meat, leading to a significant increase in both the concentration and diversity of ester compounds. The total ester content increased to 4165.39 μg/kg in the 75% substitution group. Notably, 2,4-Hexadienoic acid, ethyl ester, (E)-9-Octadecenoic acid ethyl ester and (Z,Z,Z)-9,12,15-Octadecatrienoic acid, ethyl ester were the dominant ester compounds in all samples. Notably, ethyl sorbate, ethyl oleate, and ethyl linolenate were the dominant ester compounds in all samples. The content of 2,4-Hexadienoic acid, ethyl ester reached a maximum of 1826.06 μg/kg, contributing a pleasant fruity note; the content of (E)-9-Octadecenoic acid ethyl ester reached 1804.65 μg/kg, imparting a mild fatty and oily aroma. Furthermore, ester compounds not only contribute directly to flavor perception but also act as important flavor precursors. Through hydrolysis and further degradation, they generate secondary volatile aromatic compounds including alcohols, aldehydes, furans, and lactones [20]. It is worth noting that characteristic sulfur-containing volatile compounds of sauerkraut, such as methanethiol and isothiocyanates [21], were not detected in any pork baozi filling samples. This is likely due to their extremely high volatility, substantial loss during the 20-min steaming process, and concentrations below the GC-MS detection limit.

Aldehyde compounds are primarily generated from the decomposition of alkoxy radicals during lipid oxidation, the Maillard reaction, and Strecker degradation [22]. In general, straight-chain aldehydes arise predominantly from the oxidative cleavage of unsaturated fatty acids, whereas branched-chain aldehydes originate mainly from amino acid degradation. Notably, aldehyde compounds with higher molecular weights tend to exhibit reduced olfactory pungency in meat products [23]. Under the high-temperature and high-humidity steaming conditions used in this study, lipid oxidation was the dominant pathway for aldehyde formation. Aldehyde compounds have relatively low odor thresholds [24]; even at trace concentrations, they exert a critical influence on flavor perception. They constitute the primary contributors to the characteristic aroma of pork-based fillings, a finding consistent with [25]. The content of most aldehyde flavor compounds displayed a unimodal change pattern along with the elevation of sauerkraut juice substitution level, which increased initially and declined subsequently, and the highest concentration was detected in the sample with a 75% substitution ratio. At this substitution level, a total of 32 aldehyde compounds were detected, with their combined concentration reaching 15,479.12 μg/kg. This enhancement in aldehyde formation is attributed to sauerkraut juice lowering the filling pH, which suppresses the early stages of the Maillard reaction, thereby shifting the dominant flavor formation pathway toward lipid oxidation and increasing aldehyde production from fatty acid degradation [26]. The underlying mechanism of enhanced lipid oxidation involves three interrelated factors: (1) Acid-induced structural alterations in myofibrils: The acidic environment causes partial denaturation, aggregation, or changes in the solubility of myosin filaments. These structural modifications increase the exposure of lipids, especially membrane phospholipids that are normally embedded within the sarcolemma or associated with proteins, rendering them more susceptible to oxidative damage [27]. (2) Catalytic activity of metal ions: Metal ions are potent catalysts for lipid autoxidation, and their catalytic activity is pH-dependent. Trace amounts of metal ions naturally present in sauerkraut juice accelerate lipid peroxidation (a free-radical chain reaction) when mixed with minced meat [28]. (3) pH modulation of lipoxygenase activity: Endogenous lipoxygenase exhibits optimal activity within a specific pH range. Sauerkraut juice adjusts the system pH to align more closely with this optimal range, thereby enhancing the oxidation of specific fatty acids [29].

Among these aldehydes, the major compounds with relatively high concentrations were as follows: (E,E)-2,4-Decadienal (4202.64 μg/kg), (E)-2-Dodecenal (3078.38 μg/kg), trans-4,5-Epoxy-(E)-2-decenal (1347.49 μg/kg), (E,Z)-2,4-Decadienal (1208.54 μg/kg), and Hexanal (961.87 μg/kg), among others. Hexanal imparts a characteristic green, grassy note; it is primarily formed via the decomposition of intermediate oxidation products of oleic acid, linoleic acid, and arachidonic acid, and can also arise from the degradation of certain unsaturated aldehydes. Notably, both trans-4,5-Epoxy-(E)-2-decenal and cis-4,5-Epoxy-(E)-2-decenal were detected in all experimental groups at relatively high contents. These epoxy aldehydes are formed via epoxidation of the C4-C5 double bond in (E)-2-Decenal, representing downstream products in the lipid oxidation cascade. (E)-2-Decenal itself is the primary oxidation product of unsaturated fatty acids, especially linoleic acid, under thermal and oxidative conditions. It imparts characteristic fatty and citrus-like notes and plays a critical role in the formation of the desirable cooked meaty aroma during meat thermal processing. The conversion of (E)-2-Decenal to epoxy aldehydes produces compounds with enhanced chemical reactivity and significantly intensified flavor characteristics. These epoxy aldehydes have exceptionally low odor thresholds and exhibit intense metallic, oily, and green odors even at trace concentrations, making them important marker compounds for lipid oxidation progression in meat products. The synergistic effect of acidity and metal ions in sauerkraut juice catalyzes the decomposition of hydroperoxides and promotes cyclo-oxidation reactions during lipid oxidation, thereby accelerating the formation of these epoxy aldehydes. Furthermore, (E)-2-decenal and (E)-2-dodecenal are characteristic lipid oxidation products and serve as key aroma contributors in cooked chicken, beef, and pork [30]. The high abundance of aldehyde compounds in this study is also strongly associated with the steaming processing method. In cooked pork, aldehydes are well documented as the predominant volatile class in steamed samples [31]. Compared with boiling, steaming yields a richer volatile profile and better preserves the inherent meat flavor by minimizing leaching of aroma compounds into water. Aldehydes, in particular, contribute green, sweet, citrusy, and fatty notes that substantially enhance the flavor complexity of pork baozi filling.

As another dominant class of volatile components, ketones are mainly generated via lipid oxidation and the Maillard reaction. Their odor thresholds are considerably higher than those of corresponding isomeric aldehydes [32]. Consequently, ketones are generally regarded as having minimal direct impact on overall flavor perception; instead, they function predominantly as synergistic and modulatory components that enhance the sensory profile of other volatile flavor compounds, particularly by contributing fruity and creamy notes to meat products [33]. In this study, a total of nine ketone compounds were identified, and both the diversity and concentration of detected ketones increased with the sauerkraut juice substitution level. The dominant compounds and their sensory characteristics were as follows: 5-dodecyldihydro-2(3H)-furanone (505.97 μg/kg, commonly known as γ-nonyl lactone) exhibited a characteristic coconut-like fragrance; 2-ethylcyclohexanone (395.76 μg/kg) imparted a pronounced citrus-like aroma reminiscent of orange and lemon; 1-phenyl-1-hexanone (236.54 μg/kg) had a pungent floral odor; and 2-pentadecanone (163.23 μg/kg) presented a floral and fruity aroma. This upward trend can be attributed to two factors. First, sauerkraut naturally contains ketonic compounds including 2-pyrroline and 2,5-octanedione, which impart fresh and floral aromas and directly increase the ketone content of the fillings. Second, during the steaming of minced pork, elevated temperatures promote the thermal degradation of certain long-chain ketones into smaller molecular fragments, thereby increasing the relative abundance of short-chain and heterocyclic ketones [34].

Organic acids are another important class of volatile flavor compounds in pork baozi fillings, with acetic acid being the predominant acidic component. The acetic acid content reached 662.18 µg/kg in this study, making it likely the primary contributor to the sour taste of the pork baozi fillings. Acetic acid has a low odor and taste threshold, imparting a distinct pungent sour note, and its concentration generally shows a marked increasing trend during meat processing and storage. Acetic acid in the fillings originates from three main sources: first, it is a naturally occurring volatile compound in raw pork [35]; second, it is a major organic acid component in sauerkraut [36]; third, it is generated during thermal processing via the oxidative β-scission of unsaturated fatty acid hydroperoxides, which produces short-chain fatty acids including acetic acid. (E)-9-Octadecenoic acid and n-hexadecanoic acid are the dominant free fatty acids in pork baozi fillings, accounting for 55.90% and 21.78% of total free fatty acids, respectively, a finding consistent with the subsequent fatty acid profiling results. The relatively high sugar content in sauerkraut juice may promote the formation of aldehydes, ketones and other carbonyl compounds through competing reaction pathways, diverting shared reaction precursors away from organic acid formation and ultimately reducing the total yield of organic acids. In line with this, van Boekel (2006) reported that changes in the relative proportions of substrate components, along with subsequent shifts in pH and substrate concentration, can shift the reaction product distribution from organic acids toward aldehydes [26].

Other volatile components, including alkanes, alkenes, and heterocyclic compounds, were also detected in the pork baozi fillings. Alkanes were the most abundant group in terms of compound number, dominated by long-chain alkanes at relatively low concentrations. These compounds are primarily generated from the thermal degradation of fatty acids and esters and impart a faint waxy and sweet note [37]. Nevertheless, alkanes contribute little to the overall flavor of the product owing to their high odor thresholds. Straight-chain and branched alkanes such as undecane, dodecane, tetradecane, hexadecane, heptadecane, 5-methyltridecane, and 3-methyltridecane are commonly detected volatile components in beef, pork, and fish products. Alkenes in the fillings originate from two distinct pathways: aliphatic alkenes are formed via thermal cracking and oxidative degradation of fatty acids, while terpene alkenes are primarily introduced from spices. Long-chain aliphatic alkenes generally have high odor thresholds and contribute little to the overall aroma. In contrast, terpene compounds exhibit characteristic sensory properties and play a noticeable role in flavor modulation. Anethole (1137.50 μg/kg) imparts a sweet taste and distinct anise-like aroma and is a widely used food flavor additive naturally present in fennel oil, star anise oil, and fennel seed oil. D-Limonene (76.87 μg/kg) is abundant in citrus peels, spices, and herbal essential oils, with orange peel essential oil containing the highest proportion, ranging from 42.8% to 97.3% [38]. Heterocyclic compounds, including pyrazines, pyrroles, furans, and thiophenes, are mainly formed through the Maillard reaction, Strecker degradation, and thermal degradation of free amino acids, polypeptides, and proteins [11,39]. Among them, 2-pentylfuran (342.07 μg/kg) is a well-recognized marker of lipid oxidation with a low olfactory detection threshold. It imparts a characteristic green, plant-like aroma and acts as an important volatile flavor contributor in meat products. However, due to their high volatility and chemical reactivity, heterocyclic compounds are prone to oxidation and degradation during storage and reheating, resulting in a substantial decrease in concentration over time [40].

### 3.2. OPLS-DA Analysis

Building on the qualitative and quantitative results of volatile compounds described above, orthogonal partial least squares discriminant analysis (OPLS-DA) was performed on the SPME-GC-MS dataset to further reveal the flavor profile differences among pork baozi filling samples with different sauerkraut juice substitution levels. As shown in Figure 3A, the OPLS-DA model achieved clear separation of filling samples with different substitution levels. The model exhibited excellent fit and predictive performance, with an R^2^X of 0.960, an R^2^Y of 0.993, and a Q^2^ of 0.985. Generally, R^2^Y and Q^2^ values above 0.5 indicate a reliable and well-fitted model [41]. Permutation testing (200 permutations) was further conducted to validate model robustness. The vertical intercept of the Q2 regression line was negative, demonstrating that the model had no overfitting issue and possessed favorable statistical reliability. These results collectively indicate that the model is robust and suitable for distinguishing pork baozi fillings with different sauerkraut juice substitution levels. Variable Importance in Projection (VIP) scores were calculated to quantify the contribution of each volatile component to inter-group differentiation. A higher VIP score corresponds to a more critical role of the target compound in distinguishing sample groups. Compounds with VIP > 1 are generally recognized as key differential variables driving group discrimination. The VIP scores of all detected VOCs are visualized in the heatmap shown in Figure 3C. Using a threshold of VIP > 1, a total of 19 key differential volatile flavor compounds were screened out, including (E,E)-2,4-decadienal, (E)-2-dodecenal, (E)-9-octadecenoic acid ethyl ester, oleic acid, and 2,4-hexadienoic acid ethyl ester.

### 3.3. GC-IMS Volatile Profiles

To complement the GC–MS quantification results and further characterize the volatile profiles of pork baozi fillings with high sensitivity, GC–IMS analysis was performed on samples with different sauerkraut juice substitution levels. By utilizing the built-in analysis module of the GC–IMS system software, volatile fingerprint profiles were generated to intuitively display the distribution and variation of volatile organic compounds across all samples. Each row in the profile corresponds to one sample, and each column represents a single volatile compound. The color intensity of each signal spot reflects the relative concentration of the compound, with darker color intensities correspond to elevated abundance.

As shown in Figure 4A, both the number and signal intensity of VOCs changed markedly with increasing substitution levels, confirming that sauerkraut juice alters the type and concentration of volatile compounds in pork baozi fillings. This modulation may be mainly attributed to the organic acids and phenolic constituents inherently present in sauerkraut juice, which may exert regulatory effects on lipid oxidation and the Maillard reaction, and further affect the interaction and interconversion of flavor compounds [42]. Figure 4B presents the relative difference in VOC signal intensity of treatment groups compared with the control group (0% sauerkraut juice substitution). The color gradient represents the magnitude of the concentration difference. With rising substitution levels, the diversity of VOCs increased, and the concentrations of multiple compounds were significantly elevated. For all identified VOCs across sample groups, retention times fell within 100–450 s, and ion drift times ranged from 1.0 to 2.0 ms.

A total of 66 substances were positively identified, comprising 12 esters, 15 alcohols, 11 aldehydes, 8 ketones, 4 acids, 2 alkanes, and 8 other types of compounds. Notably, esters and alcohols were more readily detected by GC–IMS, whereas aldehydes and long-chain organic acids were predominantly captured by GC–MS. This discrepancy arises from the different detection mechanisms of the two techniques: GC–IMS exhibits higher sensitivity toward small-molecule, highly volatile polar components, while GC–MS provides better coverage of semi-volatile and relatively hydrophobic compounds with larger molecular weights [43]. The combined use of the two techniques thus enables a more comprehensive characterization of the volatile flavor profile of pork baozi fillings.

Based on the signal intensity variation patterns across samples, the volatile fingerprint profile was divided into four distinct regions (A’, B’, C’, and D’), each corresponding to a characteristic concentration trend of flavor compounds with increasing sauerkraut juice substitution levels. Region A’ comprised VOCs including n-hexanol, 2-octanol, and (E)-2-heptenal. These compounds contribute desirable floral, fermented, fatty, and citrus notes to the pork baozi fillings, and their concentrations peaked at the 75% substitution level. When the substitution level further increased to 100%, their concentrations decreased. This reduction may be attributed to excessive acidity inhibiting lipid oxidation, which in turn suppresses the formation of these lipid-derived volatile substances. Compounds in Region B’, including 5-methyl-2(3H)-furanone, cyclopentanone, 2-methylbutanal, 2,3-pentanedione, and 1-propanol, followed a unimodal trend (initial increase followed by subsequent decrease) with rising sauerkraut juice substitution level, with their maximum concentrations observed at the 50% substitution level. Region C’ contained the largest number of identified compounds, including 4 aldehydes, 4 esters, 1 ketone, 2 alcohols, 1 acid, pyridine, and thienone. The concentrations of these compounds reached their maximum at the 100% substitution level. Unsaturated aldehydes, as key intermediates in the oxidative degradation of lipids, participate in reactions between amino acids and carbonyl compounds and represent the primary volatile compounds generated during pork fat oxidation [44]. Their continuous elevation at high substitution levels is likely associated with the sustained promotion of lipid oxidation by the strongly acidic environment. Region D’ was dominated by compounds with higher abundance in the control group, including 1,3-butanediol, ethyl lactate, and butyl formate. These compounds impart a mild alcoholic odor and subtle fruity aroma, and their concentrations decreased progressively with increasing substitution level. In addition, compounds including 2-butanol, 1,2-dimethoxyethane, 4-hydroxy-4-methyl-2-pentanone, and trimethylamine remained relatively stable in concentration across all sauerkraut juice substitution levels. These compounds typically exhibit strong pungent odors and are mainly derived from the spice ingredients in the filling formulation.

### 3.4. Fatty Acid Composition

To elucidate the formation mechanism of lipid-derived volatile flavor compounds presented above, the relative fatty acid composition of pork baozi fillings with different sauerkraut juice substitution levels was analyzed. As key precursors of meat flavor, fatty acids (FAs) are mainly released via the hydrolysis of phospholipids and triglycerides, and serve as core substrates for subsequent lipid oxidation to generate volatile products including aldehydes, alcohols, and ketones [44]. As shown in Figure 5, the fatty acid profile of pork baozi fillings was composed of saturated fatty acids (SFA), monounsaturated fatty acids (MUFA), and polyunsaturated fatty acids (PUFA). A total of 10 SFA, 3 MUFA, and 4 PUFA were identified. Palmitic acid (C16:0) and stearic acid (C18:0) accounted for the largest proportion of SFA species. As the sauerkraut juice substitution ratio rose, the relative content of SFA showed a trend of first increasing and then decreasing, with the lowest value found in the 100% substitution group. Meanwhile, the relative content of unsaturated fatty acids (UFA) increased gradually, indicating a potential optimization of the fatty acid nutritional profile of the fillings. (E)-9-octadecenoic acid (C18:1n9t) was the predominant MUFA component, which is an important precursor of pork characteristic aroma and exerts a critical influence on the final flavor profile of the fillings [45]. For PUFAs, arachidonic acid (C20:4n6) was the main component; it is an essential fatty acid that cannot be synthesized by the human body and must be obtained from the diet. The oxidative degradation products of n-6 PUFAs, including arachidonic acid, cover various volatile aldehydes such as hexanal and nonanal [46]. In this study, nonanal maintained a relatively high concentration (550.83 μg/kg) at high substitution levels, which was consistent with the oxidative consumption trend of arachidonic acid, and further verified the contribution of fatty acid oxidation to volatile flavor formation.

Notably, the relative content of PUFAs exhibited a unimodal trend of initial increase followed by decrease with rising sauerkraut juice substitution levels, driven by the coupled effects of lipid hydrolysis and oxidative degradation. Specifically, its relative content decreased markedly from 3.93% in the control group to 0.83% in the 100% substitution group. Concurrently, a substantial accumulation of unsaturated fatty acid ethyl esters was detected in the ester fraction, indicating extensive PUFA consumption at high substitution levels via two pathways: oxidative degradation into secondary products such as aldehydes and alcohols, and esterification with endogenous alcohols to form fatty acid ethyl esters [47]. The pH reduction induced by sauerkraut juice drove the staged evolution of lipid metabolism in the fillings. At low substitution levels, lipid oxidation remained in the initial stage. Under the weakly acidic condition, endogenous acidic phospholipases and lipases were activated, which accelerated the hydrolysis of phospholipids and triglycerides and promoted the release of free fatty acids [48]. At this stage, meat-derived volatile compounds dominated the overall flavor profile. With a further decrease in pH, phospholipid hydrolysis was intensified, and free PUFA content reached its peak, providing abundant substrates for subsequent oxidation reactions and leading to the accumulation of large amounts of primary lipid oxidation products. As pH continued to decline at the 100% substitution level, PUFAs underwent oxidative consumption, and a large quantity of volatile oxidation intermediates were generated. Notably, however, these newly formed volatiles did not accumulate proportionally in the headspace. Instead, the high concentrations of phenolics and vitamin C introduced at 100% substitution promoted secondary reactions among the volatile oxidation products, partially consuming these compounds. In general, the relative PUFA content was significantly higher in the low-to-medium substitution groups than in the control group; by contrast, a declining trend was detected in the 100% substitution group.

### 3.5. E-Nose Odor Profiles

To assess the overall olfactory differences among pork baozi fillings with different sauerkraut juice substitution levels, electronic nose (E-nose) analysis was conducted. The E-nose achieves sample discrimination and classification based on the differential response patterns of its sensor array to specific VOCs [49]. Radar plots of E-nose sensor response values for all samples are shown in Figure 6A. Overall, the five groups of fillings shared similar flavor profile contours but differed notably in sensor response intensity. Compared with the control group, most sensors showed significantly elevated response values after sauerkraut juice addition, and the response intensity peaked at the 75% substitution level, which was consistent with the GC–MS quantification results. Sensors S1, S5, S11, S12, S20, S25, and S26 exhibited the most pronounced variation across groups, indicating that the VOCs driving flavor differences were mainly compounds recognized by these sensors, including short-chain alkanes, nitrates, allyl sulfides, ketones and alcohols, amines, sulfur-containing odorants, organic acid esters, and terpenes. This result aligned well with the GC–MS findings, which detected high concentrations of 2,4-hexadienoic acid ethyl ester, acetic acid, 2-hydroxy-3-acetyl-6-methyl-4-pyrone, nitroso-methane and estragole. In raw pork, the predominant VOCs include acetic acid, acetoin, 1,3-dimethylbenzene, methyl acetate, ethanol, and limonene [35]. Raw Chinese cabbage has an inherent pungent odor, mainly derived from VOCs such as methyl thiocyanate and allyl isothiocyanate [50]. During fermentation, sulfides and sour aroma compounds are generated; acetic acid and ethyl acetate are the core contributors to the sour aroma profile, which may account for the significant response increase of sensors S11, S20, and S26.

To further quantify the olfactory differences among samples, principal component analysis (PCA) was performed on the E-nose data, with the results shown in Figure 6B. PC1 and PC2 accounted for 83.6% and 9.9% of the total variance, respectively, with a cumulative explained variance of 93.5%. This indicates that the PCA model effectively captured the overall flavor characteristics of each group and enabled clear discrimination among the five filling groups. Sample points from the 50% and 75% substitution groups clustered closely together, indicating similar odor profiles between these two groups, and they were clearly separated from the other three groups. This observation was consistent with the trend shown in the radar plots.

### 3.6. E-Tongue Taste Profiles

Following the olfactory characterization via E-nose, electronic tongue (E-tongue) analysis was conducted to evaluate the gustatory profiles of pork baozi fillings with different sauerkraut juice substitution levels. The E-tongue utilizes cross-selective sensor arrays to simulate human gustatory perception and quantitatively characterize taste attributes of samples. The aftertaste and overall taste richness of meat products are closely associated with the contents of small peptides and free amino acids [51]. As shown in Figure 6C, both umami intensity and taste richness of the fillings exhibited first decreasing and then increasing as the sauerkraut juice substitution ratio increased. At the 75% and 100% substitution levels, umami intensity recovered to the level of the control group, while taste richness was significantly higher than that of the control. In contrast, the response values of bitterness and astringency showed a marked downward trend with increasing substitution levels, indicating that sauerkraut juice effectively attenuated the unfavorable taste attributes of the fillings. This reduction can be explained by three complementary mechanisms. First, the complex flavor matrix of sauerkraut juice exerts a sensory masking effect on bitter and astringent perception. Second, the decreased system pH induces partial protein denaturation, which reduces the generation of bitter peptides and further weakens their binding to gustatory receptors on the tongue [52]. Third, differential protein solubility during sample pretreatment also contributes to the reduced readings. As the substitution level of acidic sauerkraut juice increases, the pH approaches the isoelectric point of meat proteins, driving marked increases in protein coagulation and insolubilization. Consequently, high-substitution samples are likely to have a much larger fraction of proteins and peptides physically filtered out before reaching the sensors compared with the control group, resulting in lower response values.

Principal component analysis was performed on the E-tongue data to visualize the overall taste differences among samples, with the results presented in Figure 6D. PC1 and PC2 accounted for 42.3% and 35.1% of the total variance, respectively, with a cumulative variance contribution rate of 77.4%. Samples with different sauerkraut juice substitution levels were relatively scattered on the PCA score plot and were clearly distinguished from the control group. PC1 played a dominant role in explaining the variance of sample taste information, indicating that taste differences among samples could be mainly distinguished along the horizontal axis.

### 3.7. Sensory Properties

To correlate the instrumental olfactory and gustatory analyses with human perceptual experience, descriptive sensory evaluation was performed on pork baozi fillings with different sauerkraut juice substitution levels. As shown in Figure 6E, partial substitution of water with sauerkraut juice exerted no significant influence on the appearance and texture of the fillings, but markedly modulated their overall flavor profiles. Fillings prepared with the 75% substitution level received the highest overall sensory quality scores, with optimal flavor performance and well-balanced sourness. One plausible explanation is that at this substitution ratio, flavor precursors introduced by sauerkraut juice (amino acids, sugars, and phenolic compounds) may achieve an optimal balance with the endogenous components of the meat matrix. This creates favorable conditions for the generation of key aroma-active compounds and may maximally promote core flavor-forming pathways, including the Maillard reaction and lipid oxidation. However, at the 100% substitution level (complete replacement of water), the fillings exhibited prominent sour and pungent notes inherent to sauerkraut juice, which overwhelmed the inherent meaty aroma and led to a decline in overall sensory quality score.

## 4. Conclusions

This study systematically explored the dynamic variations in the volatile flavor profiles of pork baozi fillings prepared by substituting processing water with sauerkraut juice at different levels. The overall diversity and concentration of VOCs exhibited a unimodal trend (initial increase followed by decrease) with rising substitution levels, reaching the maximum at the 75% substitution level. This unimodal pattern may arise from the coupled effects of pH-mediated lipid oxidation promotion and phenolic/vitamin C–driven secondary consumption of volatile products. Using a combination of GC–MS and GC–IMS, a total of 80 and 66 volatile compounds were identified, respectively. The complementary advantages of the two techniques allowed for a more comprehensive characterization of the dynamic changes in VOCs. Furthermore, the OPLS–DA model combined with VIP analysis screened out 19 key differential flavor compounds that contributed most to inter-group flavor differences, including (E,E)-2,4-decadienal, (E)-2-dodecenal and 2,4-hexadienoic acid ethyl ester. These compounds can serve as potential markers for flavor quality evaluation of pork baozi fillings. Consistent with the compositional analysis, results from E-nose, E-tongue and descriptive sensory evaluation collectively confirmed that the 75% substitution level achieved the optimal flavor and taste performance, with well-balanced sourness and the highest overall sensory quality score. Moderate addition of sauerkraut juice effectively improved the flavor complexity and taste richness of pork baozi fillings without impairing their appearance and texture attributes. These findings provide a systematic theoretical basis for elucidating the flavor enhancement mechanism of sauerkraut juice in pork baozi fillings, and offer objective data support for product development and quality control of sauerkraut-stuffed steamed buns and related meat products.

## Figures and Tables

**Figure 1:**
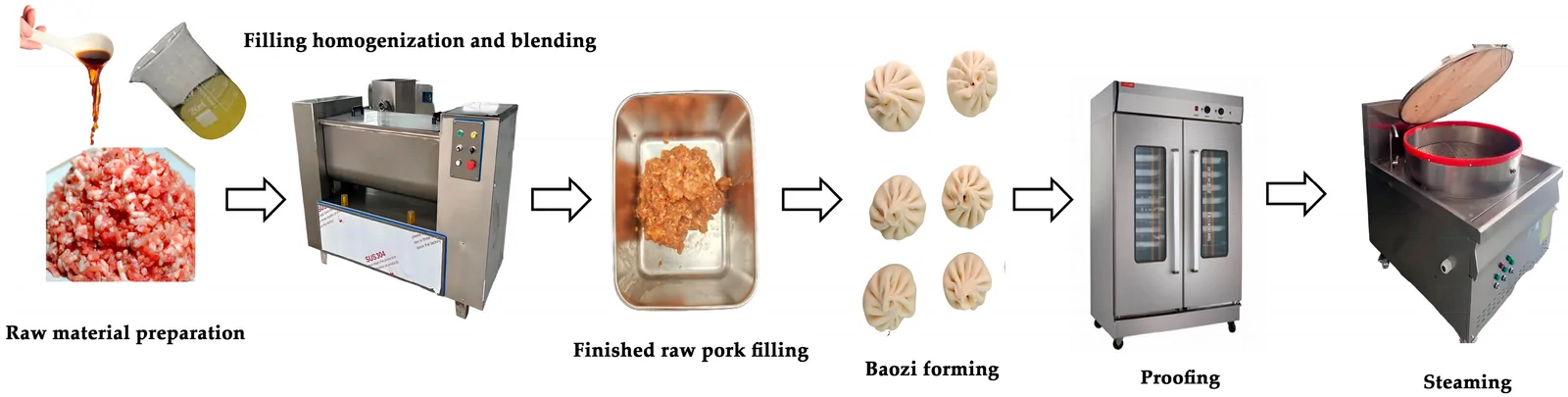
Process flow diagram of pork baozi preparation.

**Figure 2:**
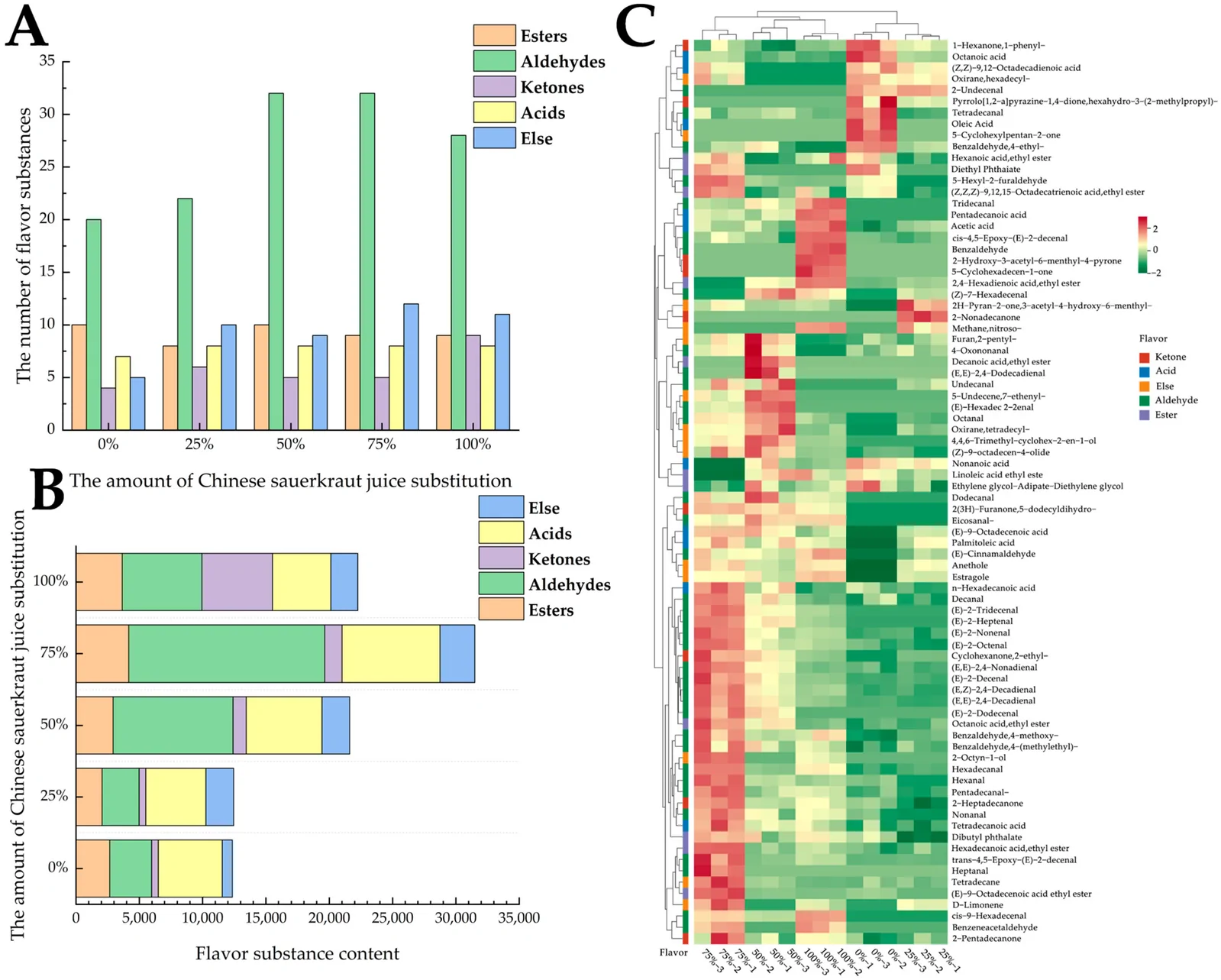
Characterization of volatile flavor compounds in pork baozi fillings: (**A**) variation profile of volatile flavor compounds, (**B**) content variation of volatile flavor compounds, and (**C**) clustered heatmap of volatile flavor compounds.

**Figure 3:**
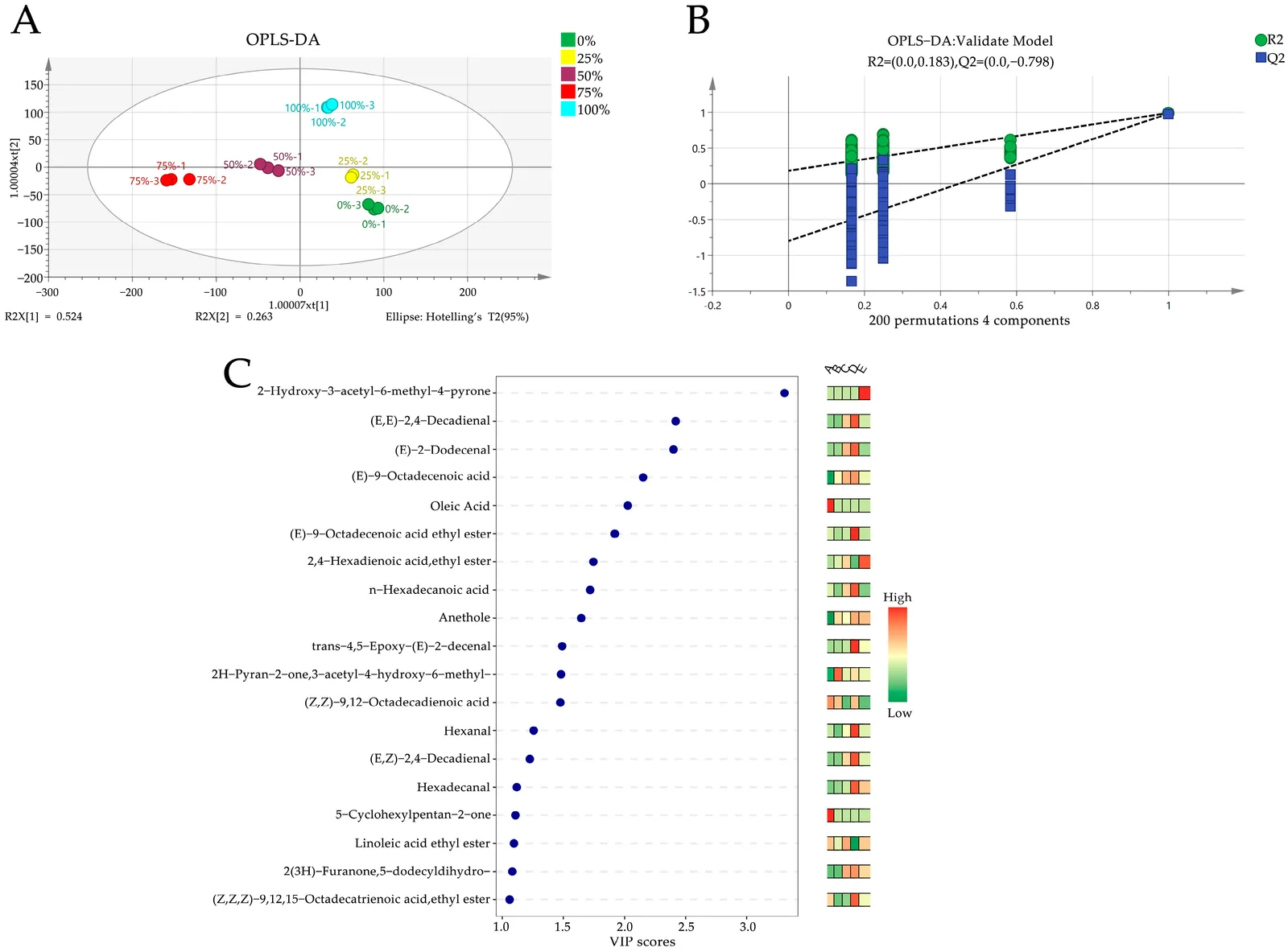
OPLS-DA model of volatile compounds in pork baozi fillings: (**A**) OPLS-DA score plot of different samples, (**B**) results of 200 permutation tests, and (**C**) VIP values of volatile compounds. (A, B, C, D, and E represent sauerkraut juice substitution levels of 0%, 25%, 50%, 75%, and 100%, respectively).

**Figure 4:**
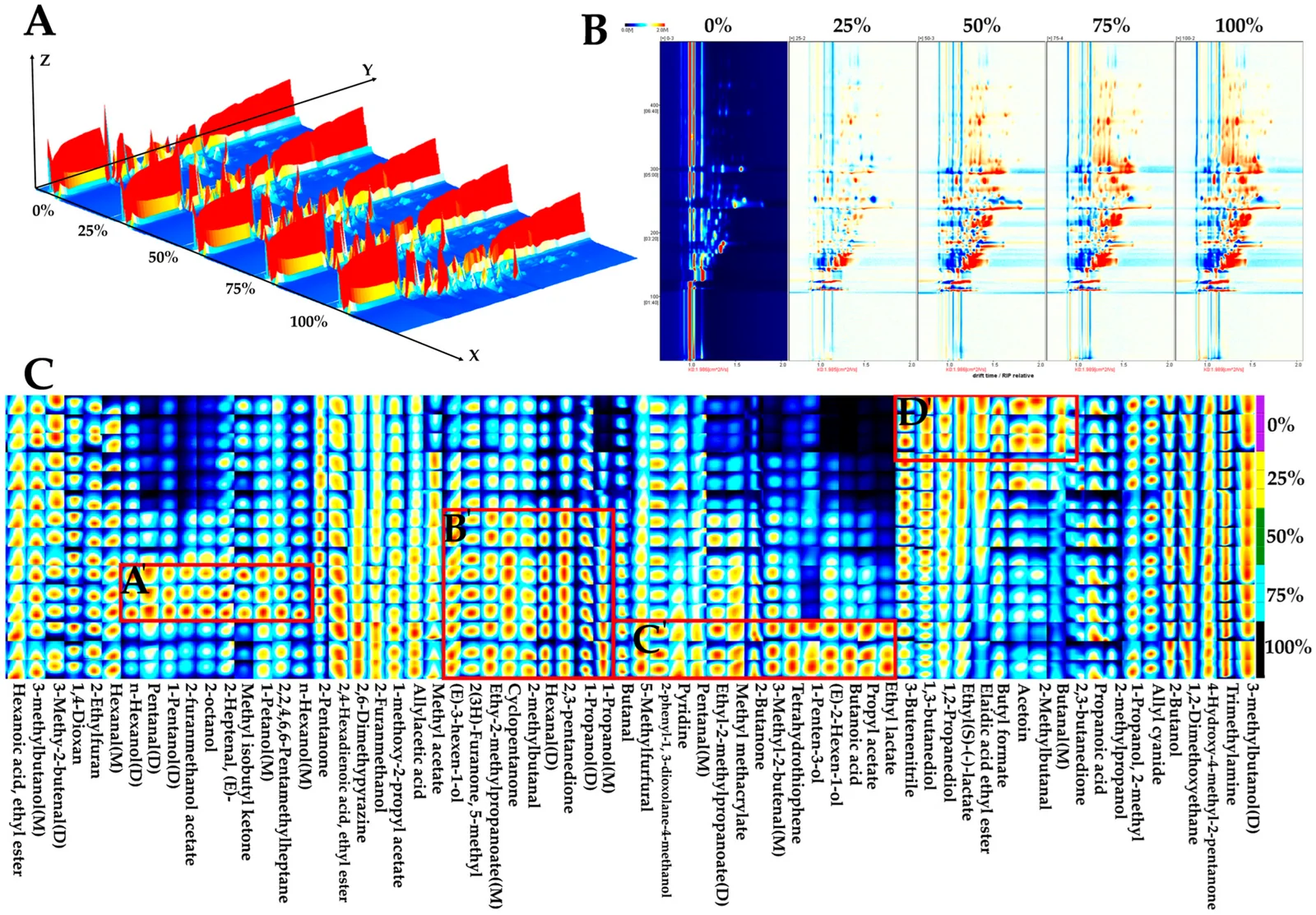
Analysis of volatile flavor compounds in pork baozi fillings by GC-IMS: (**A**) 3D topographic plot, (**B**) 2D topographic plot, and (**C**) volatile compound fingerprints. Note. The 3D topographic plot clearly reveals variations in the types and concentrations of VOCs in pork baozi fillings at different sauerkraut juice substitution levels. Each signal peak corresponds to a specific volatile compound. The X-, Y-, and Z-axes correspond to drift time, retention time, and signal intensity, respectively. The red vertical line at an *X*-axis value of 1.0 denotes the normalized reaction ion peak (RIP). Each colored spot reflects the signal intensity of a specific VOC, with red and white indicating high and low intensity, respectively. A given compound may appear as one, two, or more distinct spots, corresponding to its monomer, dimer, or trimer forms, depending on its concentration and physicochemical properties.

**Figure 5:**
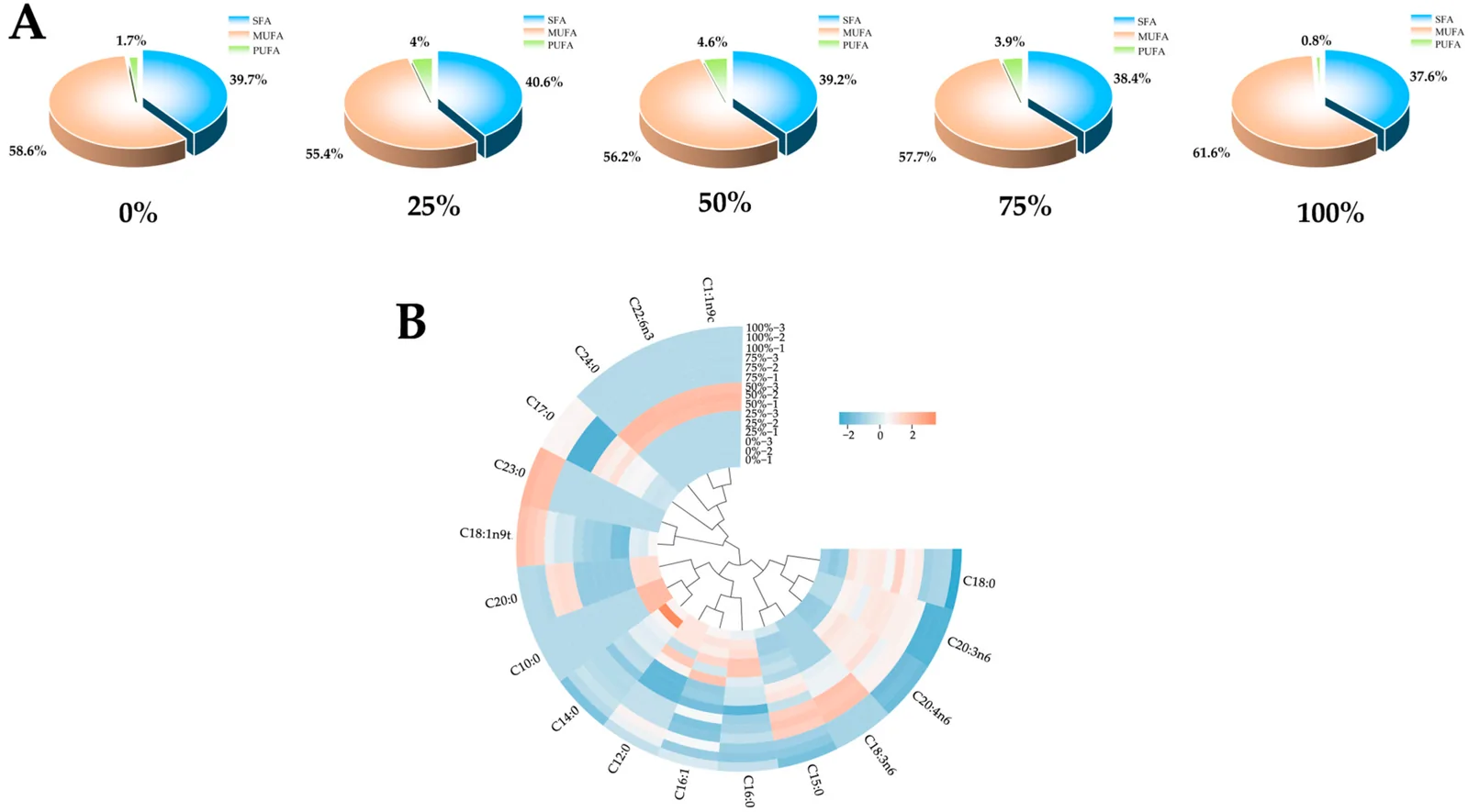
Analysis of fatty acid composition in pork baozi fillings: (**A**) variation in fatty acid content as a function of sauerkraut juice substitution level, and (**B**) heatmap of fatty acid composition.

**Figure 6:**
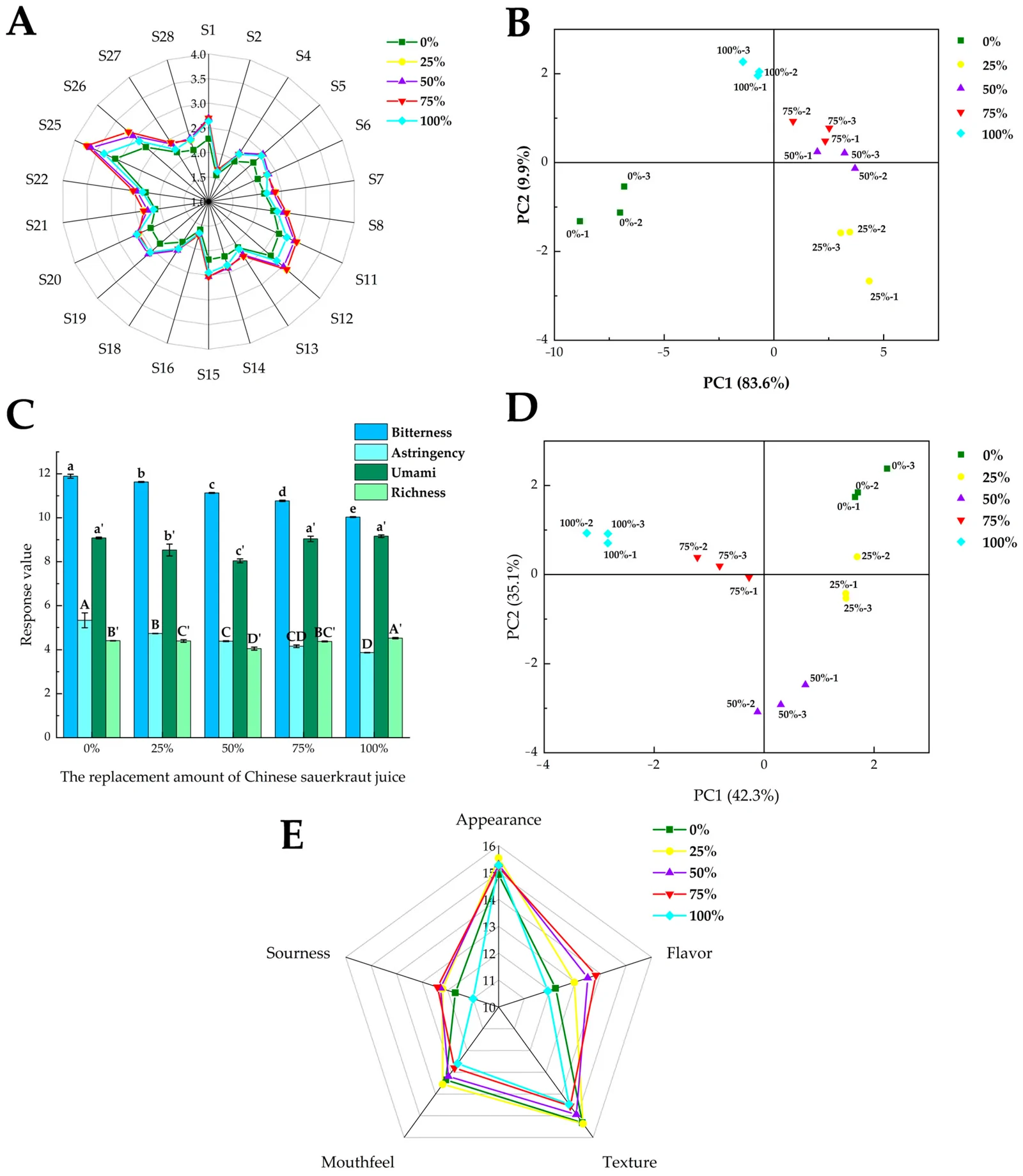
Sensory evaluation of pork baozi fillings with different sauerkraut juice substitution levels: (**A**) radar chart of electronic nose response values, (**B**) PCA score plot of electronic nose response values, (**C**) bar chart of electronic tongue response values, (**D**) PCA score plot of electronic tongue response values, and (**E**) radar chart of sensory evaluation scores. Note. Different lowercase letters (a’–c’) and (A’–C’) indicate significant differences among groups for the same indicator (*p* < 0.05).

## Data Availability

The original contributions presented in the study are included in the article, further inquiries can be directed to the corresponding author.
